# Associations between sensitivity to thyroid hormones and insulin resistance in euthyroid adults with obesity

**DOI:** 10.3389/fendo.2024.1366830

**Published:** 2024-08-08

**Authors:** Ying Wei, Xingang Li, Ruixiang Cui, Jia Liu, Guang Wang

**Affiliations:** Department of Endocrinology, Beijing Chao-Yang Hospital, Capital Medical University, Beijing, China

**Keywords:** thyroid hormone sensitivity, insulin resistance, adipose tissue, obesity, metabolic diseases

## Abstract

**Background:**

Impaired sensitivity to thyroid hormones (TH) was associated with metabolic syndrome. The study aimed to explore the association between central TH sensitivity indices and insulin resistance (IR) in euthyroid adults with obesity.

**Methods:**

This cross-sectional study enrolled 293 euthyroid outpatients with obesity in Beijing Chao-Yang Hospital. We used the thyroid feedback quantile-based index (TFQI), thyroid stimulating hormone index (TSHI), and thyrotrophic T4 resistance index (TT4RI) to indicate central TH sensitivity. IR was assessed by homeostasis model assessment of insulin resistance (HOMA-IR), hepatic insulin resistance index (hepatic-IR), the Matsuda index, and the adipose tissue insulin resistance index (Adipo-IR). Participants were categorized according to tertiles of TH sensitivity indices. We used multiple linear regressions to explore the associations.

**Results:**

There was a significant stepwise increase in HOMA-IR and Adipo-IR from the lowest to the highest tertiles of TH sensitivity indices (all *P<*0.05). After adjustment for age, sex, body mass index, hypertension, hyperlipidemia, and diabetes, only Adipo-IR was significantly associated with TH sensitivity indices. On average, each unit increased in TFQI, TSHI, and TT4RI was associated with 1.19 (*P*=0.053), 1.16 (*P*=0.04), and 1.01 (*P*=0.03) units increased in Adipo-IR, respectively. There was no significant association between TH sensitivity indices and HOMA-IR, hepatic-IR, and the Matsuda index after adjustment for other risk factors.

**Conclusions:**

Reduced central TH sensitivity was associated with increased adipose tissue insulin resistance in euthyroid adults with obesity. The results further confirmed the importance of TH sensitivity on metabolic diseases.

## 1 Introduction

Evidence for the association between thyroid hormones (THs) and glucose metabolism appeared for a long time. THs have an insulin-antagonistic effect on the liver, leading to increased hepatic glucose output by enhancing the rate of gluconeogenesis and glycogenolysis (1). Thyroid stimulating hormone (TSH) and THs correlate with multiple cardiometabolic risk factors, including insulin resistance (IR) (2). Subjects with hyperthyroidism or hypothyroidism can also show impaired glucose tolerance and IR (1). Dysregulation of carbohydrate metabolism and thyroid dysfunction are closely linked. TH can regulate insulin secretion by both direct and indirect pathways, which may involve diminishing the secretion triggered by glucose or attenuating the sensitivity of pancreatic β cells (3). However, studies addressing the association between THs and IR have been controversial. Jose’s study indicated that both serum TSH and free thyroxine (FT4)*TSH product were positively associated with IR in healthy euthyroid subjects without diabetes (4). Another study of 940 euthyroid participants suggested that high free tri-iodothyronine (FT3) levels within the normal range are associated with IR measured by the insulin clamp technique both cross-sectionally and longitudinally (5). Data from two independent epidemiological studies also confirmed a consistent association between FT3 and IR (6). Ambrosi’s study of 581 euthyroid subjects with obesity showed a positive association between TSH and IR but a negative association between FT4 and IR [IR was calculated by homeostasis model assessment of insulin resistance (HOMA-IR) and Quantitative Insulin Sensitivity Check Index (QUICKI)] (7). However, a Mendelian randomization study by Maxime found no evidence for a causal association between circulatory levels of TSH and FT4 with IR (8), so did another study from the sixth Korean National Health and Nutrition Examination Survey, suggesting no association between TSH, FT4, and HOMA-IR (9). In a euthyroid population with 1275 participants of obesity, TSH has no association with IR indicated by HOMA-IR, and the positive relationship between FT3 and IR lost its significance after adjustment for other confounders (10). Amouzegar’s cross-sectional study of 2758 euthyroid subjects free of thyroid disorders and diabetes found the association between THs and IR differed by sex, with no association between FT4 or TSH and HOMA-IR among women, but a positive association between FT4 and HOMA-IR among men (11). It is reasonable to assume that the discrepancies in these findings may stem from variable TH sensitivity among different populations.

The hypothalamic-pituitary-thyroid axis maintains the equilibrium of TH levels. Central sensitivity to THs reflects the pituitary responsiveness to them. Recently, researchers proposed several indices assessed by measuring circulating FT4 values and TSH values and representing the central sensitivity of TH. These indexes include thyroid feedback quantile-based index (TFQI), TSH index (TSHI), and thyrotrophic T4 resistance index (TT4RI) (12, 13). Previous studies indicated that impaired sensitivity to TH was associated with metabolic syndrome, diabetes, diabetes-related mortality, hyperuricemia, hyperhomocysteinemia, high remnant cholesterol levels, hypertension, and cardiovascular disease risk score (12, 14–17), suggesting that decreased sensitivity to THs can contribute to metabolic disorders. However, little is known about the association between the sensitivity of TH and IR, which is also a hallmark of the metabolic syndrome (18), with only one study of 80 prepubertal euthyroid children with obesity from three Italian pediatric endocrinology centers revealing that TFQI was negatively associated with the Matsuda-index (19).

THs exert bidirectional effects on insulin signaling, agonistic in muscle tissue and antagonistic in the liver. Hyperthyroidism disrupts this equilibrium, precipitating hepatic insulin resistance and glucose intolerance. Hypothyroidism induces more nuanced insulin resistance, primarily in peripheral tissues. This resistance could stem from impaired mitochondrial oxidative phosphorylation and reduced blood perfusion in muscular and adipose tissues (1). Multiple methods and indices have been refined to evaluate IR in different tissues, reflecting both static measures and dynamic testing. The intricate interplay of glucose and insulin metabolism is driven by diverse stimuli in several tissues. HOMA-IR is a simple, minimally invasive and widely used method for IR (20). The hepatic insulin resistance (hepatic-IR) index coming from the measurement of plasma glucose and insulin concentrations during an oral glucose tolerance test (OGTT) is presented for quantitation of hepatic insulin sensitivity (21). The Matsuda index is used to measure the composite whole-body insulin sensitivity including both hepatic and peripheral tissue insulin sensitivity (22). The adipose tissue insulin resistance index (Adipo-IR index) is calculated as the product of the fasting insulin and free fatty acid (FFA) concentration and is useful in large-scale clinical practice (23).

Considering the complex effect of THs on glucose metabolism and metabolic disorders, we aimed to explore the relationship between sensitivity to TH and IR in euthyroid adults with obesity. We used the following indices to assess insulin resistance of different tissues, including HOMA-IR, hepatic-IR, Adipo-IR, and the Matsuda index for whole-body insulin sensitivity.

## 2 Materials and methods

### 2.1 Study population

This cross-sectional study enrolled outpatients with obesity counseling weight loss in the Department of Endocrinology, Beijing Chao-Yang Hospital between April 2017 and August 2021. The inclusion criteria were: (1) age ≥ 18 years old; (2) obesity which is defined as a body mass index (BMI) of 28kg/m^2^ or higher in the Chinese population (24); (3) normal blood levels of TSH, FT4 and FT3 (the laboratory normality reference ranges were 0.55 - 4.78 μIU/mL for TSH, 11.45 - 22.65 pmol/L for FT4, and 2.0-4.4pg/ml for FT3); (4) receiving a 75-g OGTT. Subjects who met the following criteria were excluded: (1) missing data in FT4, FT3, TSH, FFA, or OGTT results; (2) previous history of thyroid diseases; (3) antithyroid therapy or thyroid hormone replacement treatment; (4) Elevated levels of anti-thyroid peroxidase (anti-TPO) or anti-thyroglobulin (anti-TG) antibodies (anti-TPO ≥ 60U/ml or anti-TG ≥ 60U/ml); (5) taking hypoglycemic or lipid-lowering medications; (6) severe renal or liver dysfunction (denoting as estimated glomerular filtration rate (eGFR)< 60 ml/min/1.73m2, alanine transaminase (ALT) or aspartate transaminase (AST) more than three times the upper limit of normal range). At last, the study enrolled 293 participants in the analysis. Referring to the results of previous research enrolling 80 participants and studying the association between TH sensitivity and IR (19), we assume that a sample size of approximately 300 individuals has sufficient power to detect differences. The study was in accordance with the Declaration of Helsinki and was approved by the Ethical Review Board at Beijing Chao-Yang Hospital, Capital Medical University (Approval number: 2022-Science-517). We got informed consent from all subjects.

### 2.2 Measurement of clinical information

Qualified physicians regularly collected the medical information of all participants, including age, sex, weight, height, and medical history. Venous blood samples were collected from the participants in the morning after more than 10 hours of overnight fasting, then sent to the standard clinical laboratories of Beijing Chao-Yang Hospital. We use the glucose oxidase method to measure blood glucose, the chemiluminescence method for blood insulin, and the colorimetric enzymatic method for ALT, AST, creatinine, lipids, and FFA (Siemens Healthcare Diagnostics). TSH, FT4, and FT3 were measured by electrochemiluminescence immunoassay, and anti-TPO and anti-TG were measured by chemiluminescent immunoassay, using an Abbott Architect i2000 (Abbott Diagnostics). At 8:00 A.M., after a more than 10-hour overnight fast, subjects received OGTT. Blood samples were taken at 0, 60, and 120 minutes for the measurement of blood glucose and insulin concentrations. Blood samples at 30 minutes during OGTT were also collected from 152 patients (111 patients without diabetes and 41 patients with diabetes).

### 2.3 Definition of variables

BMI is calculated by a person’s weight in kilograms divided by the square of height in meters. Dyslipidemia was defined as total cholesterol (TC) ≥ 5.2mmol/L, or triglyceride (TG) ≥ 1.7mmol/L, or low-density lipoprotein cholesterol (LDL-C) ≥ 3.4mmol/L, or high-density lipoprotein cholesterol (HDL-C)< 1.0mmol/L, or non-high-density lipoprotein cholesterol (non-HDL-C) ≥ 4.1mmol/L (25). The criteria for the diagnosis of diabetes were: fasting blood glucose (FBG) ≥ 7.0 mmol/L or hemoglobin A1C (HbA1c) ≥ 6.5% (48 mmol/mol) or random plasma glucose ≥11.1 mmol/L with classic symptoms of hyperglycemia, or taking glucose-lowering drugs (26). TFQI was calculated as cumulative distribution function (cdf) (FT4) - [1 – cdf (TSH)], i.e., the difference between the FT4 quantile and the reversed TSH quantile. This index ranges between -1 and 1, with negative values indicating higher sensitivity to FT4, and positive values indicating lower sensitivity to FT4 (12). TSHI was calculated as ln TSH (mIU/L) + 0.1345 * FT4(pmol/L) (13). TT4RI was calculated as FT4 (pmol/L) * TSH (mIU/L) (12). Higher TSHI or TT4RI represented lower central sensitivity to thyroid hormones. HOMA-IR, which has been used widely as an indirect method for quantifying insulin resistance, is defined as [FBG (mmol/L) × fasting insulin (uIU/ml))/22.5] (20). The hepatic-IR index was defined as the product of the total area under curve (AUC) for glucose and insulin during the first 30 min of the OGTT (glucose_0–30_[AUC] * insulin_0–30_[AUC]) (21). The whole-body insulin sensitivity was estimated by the Matsuda index derived from the OGTT (22):


$$\frac{10000}{\sqrt{\left(fasting\;glucose*fasting\;insulin)*(mean\;glucose*mean\;insulin\;during\;OGTT\right)}}$$

Adipo-IR index was calculated as the product of the fasting insulin and FFA concentration [Adipo-IR index = fasting insulin (μIU/mL) × fasting FFA (mmol/L)] (23).

### 2.4 Statistical analysis

We described continuous variables that fit normal distribution as mean ± standard deviation (SD), and those that didn’t conform to a normal distribution as median (quartiles), and categorical variables as count (percentage). Non-normally distributed variables were log-transformed when put into linear regression models (e.g., age, BMI, HOMA-IR, hepatic-IR, the Matsuda index, and Adipo-IR). We categorized TFQI, TSHI, and TT4RI into three groups according to tertiles (**Supplementary Table 1**), using the lowest tertile range (Q1) as the reference group. We used one-way analysis of variance (ANOVA) to compare the distributions of insulin resistance indices (after log-transformed) across tertiles of TH sensitivity indices in the crude analysis. We used multiple linear regression models to explore the association between TH sensitivity indices and insulin resistance indices after adjustment for potential confounders (e.g., age, sex, BMI, hypertension, hyperlipidemia, diabetes, eGFR). *P*< 0.05 was considered statistically significant. Analyses were carried out using R version 4.1.2.

## 3 Results

### 3.1 Baseline information

In total, there were 293 participants enrolled in the study. The median age of the total subjects was 31 years old, and the median BMI was 38.4 kg/m^2^. Most of the participants were female (71.7%), without hypertension (70.6%), without diabetes (75.1%) and with hyperlipidemia (71.3%). Results of other laboratory tests, TH sensitivity indices, and insulin resistance indices are shown in **Table 1**.

**Table 1:** Baseline information of the total subjects.

	Male (n=83)	Female (n=210)	Overall (n=293)
**Age (years)**	32 [27.5, 37.5]	31 [26, 36.8]	31 [27, 37]
**BMI (kg/m^2^)**	42.9 [36.8, 48.6]	36.8 [33.5, 42.2]	38.4 [34.1, 44.8]
**Hypertension, n (%)**	35 (42.2%)	51 (24.3%)	86 (29.4%)
**Hyperlipidemia, n (%)**	72 (86.7%)	137 (65.2%)	209 (71.3%)
**Diabetes, n (%)**	24 (28.9%)	49 (23.3%)	73 (24.9%)
**FFA (mmol/L)**	0.68 [0.54, 0.84]	0.63 [0.48, 0.78]	0.64 [0.49, 0.80]
**FT4 (pmol/L)**	16.09 [14.29, 17.12]	15.70 [14.29, 16.86]	15.83 [14.29, 16.99]
**FT3 (pg/ml)**	3.50 [3.18, 3.71]	3.19 [2.97, 3.41]	3.26 [3.02, 3.56]
**TSH (μIU/mL)**	2.41 [1.67, 3.13]	2.43 [1.99, 3.14]	2.42 [1.90, 3.14]
**TFQI**	-0.05 [-0.31, 0.38]	0.02 [-0.28, 0.31]	0.02 [-0.29, 0.33]
**TSHI**	2.92 [2.61, 3.32]	3.00 [2.72, 3.33]	2.98 [2.70, 3.33]
**TT4RI**	39.7 (16.6)	39.6 (14.5)	39.7 (15.1)
**Fasting glucose (mmol/L)**	5.65 [5.30, 6.56]	5.62 [4.98, 6.34]	5.63 [5.10, 6.40]
**0.5-hour glucose (mmol/L)**	10.12 [9.22, 11.42]	10.05 [8.58, 11.59]	10.08 [8.71, 11.57]
**1-hour glucose (mmol/L)**	10.32 [8.40, 13.04]	10.26 [8.77, 13.26]	10.27 [8.71, 13.17]
**2-hour glucose (mmol/L)**	7.87 [5.88, 10.18]	7.81 [6.52, 10.30]	7.82 [6.32, 10.27]
**Fasting insulin (uIU/ml)**	31.35 [22.96, 44.65]	25.14 [18.44, 37.64]	26.94 [18.89, 38.84]
**0.5-hour insulin (uIU/ml)**	132.2 [96.6, 193.3]	113.3 [72.3, 182.5]	117.9 [73.3, 182.9]
**1-hour insulin (uIU/ml)**	149.2 [96.5, 251.7]	133.6 [80.3, 201.9]	138.0 [82.4, 216.7]
**2-hour insulin (uIU/ml)**	108.9 [48.0, 180.7]	111.3 [64.1, 181.5]	110.7 [57.3, 182.3]
**HOMA-IR**	8.64 [6.02, 13.45]	6.42 [4.18, 10.33]	6.87 [4.36, 10.95]
**Hepatic-IR**	158.6 [126.1, 254.3]	149.3 [100.6, 206.9]	150.1 [102.6, 209.1]
**Matsuda index**	25.7 [17.4, 37.1]	29.2 [20.1, 42.9]	27.6 [19.0, 42.2]
**Adipo-IR**	21.4 [12.9, 32.6]	15.7 [9.9, 25.1]	17.5 [10.6, 27.3]

BMI, body mass index; FFA, free fatty acid; FT4, free thyroxine; FT3, free tri-iodothyronine; TSH, thyroid stimulating hormone; TFQI, Thyroid Feedback Quantile-based Index; TSHI, TSH index; TT4RI, thyrotrophic T4 resistance index; HOMA-IR, homeostasis model assessment of insulin resistance; Hepatic-IR, hepatic insulin resistance index; Adipo-IR, adipose tissue insulin resistance index.

### 3.2 Univariate analysis between TH sensitivity indices and IR

**Figure 1:**
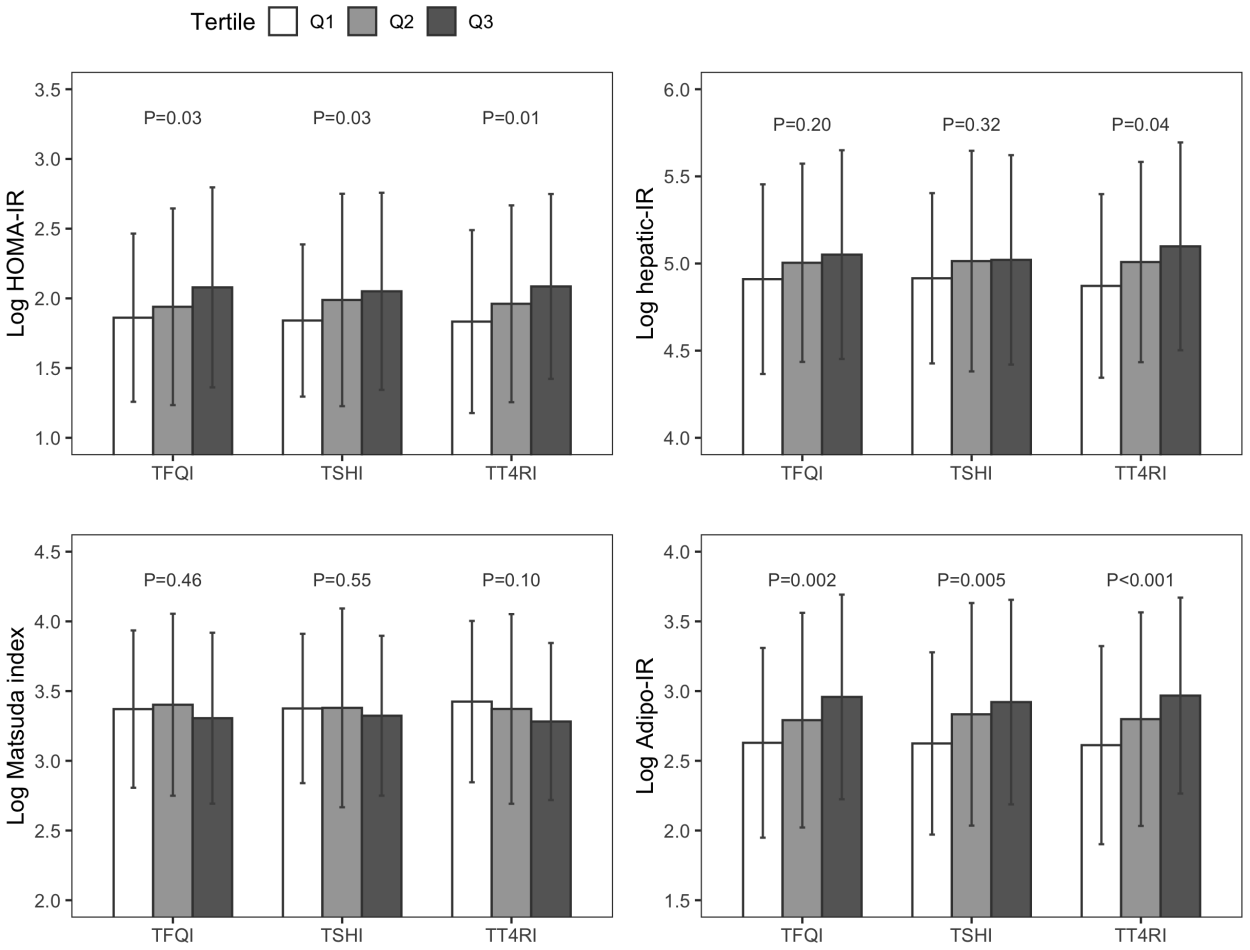
Insulin resistance indices across tertiles of thyroid hormone sensitivity indices. TFQI, Thyroid Feedback Quantile-based Index; TSHI, thyroid stimulating hormone index; TT4RI, thyrotrophic T4 resistance index; HOMA-IR, homeostasis model assessment of insulin resistance; Hepatic-IR, hepatic insulin resistance index; Adipo-IR, adipose tissue insulin resistance index.

As **Figure 1**, **Supplementary Table 2** showed, there was a significant stepwise increase in HOMA-IR and Adipo-IR from the lowest to the highest tertiles of TH sensitivity indices (all P<0.05). There were 141 (48.1%) missing data in 0.5-hour glucose, 0.5-hour insulin levels, as well as in Hepatic-IR. Hepatic-IR also increased across tertiles of TT4RI (*P*=0.04). There was no significant difference in the hepatic-IR index across tertiles of TFQI and TSHI, and the Matsuda index across tertiles of all three TH sensitivity indices (all P>0.05).

### 3.3 Multiple regression analysis between TH sensitivity indices and IR

As **Table 2** displayed, after adjustment for age, sex, BMI, hypertension, hyperlipidemia, and diabetes, only Adipo-IR was significantly associated with TH sensitivity indices. Subjects in the highest tertile of TFQI had 0.20 units increased in log Adipo-IR (e^0.20^ = 1.22) compared with subjects in the lowest tertile of TFQI (*P*=0.03). Subjects in the highest tertile of TSHI had 0.18 units increased in log Adipo-IR (e^0.18^ = 1.20) compared with subjects in the lowest tertile of TSHI (*P*=0.04). Subjects in the highest tertile of TT4RI had 0.22 units increased in log Adipo-IR (e^0.22^ = 1.25) compared with subjects in the lowest tertile of TT4RI (*P*=0.02). On average, each unit increased in TFQI, TSHI, and TT4RI was associated with 1.19 (e^0.17^=1.19, *P*=0.053), 1.16 (e^0.15^=1.16, *P*=0.04), and 1.01 (e^0.005^=1.01, *P*=0.03) units increased in Adipo-IR, respectively. Generally, there was no significant association between TH sensitivity indices and HOMA-IR, hepatic-IR, and the Matsuda index after adjustment for other risk factors.

**Table 2:** Multiple linear regression between thyroid hormone sensitivity indices and insulin resistance.

	Tertiles	TFQI		TSHI		TT4RI	
		β (SE)	*P*	β (SE)	*P*	β (SE)	*P*
HOMA-IR							
TH indices categorized	Q1	Ref	–	Ref	–	Ref	–
	Q2	0.05(0.08)	0.57	0.14(0.08)	0.08	0.09(0.25)	0.25
	Q3	0.10(0.08)	0.21	0.11(0.08)	0.19	0.17(0.08)	0.03*
TH indices continuous	–	0.09(0.08)	0.24	0.10(0.07)	0.14	0.004(0.002)	0.10
Hepatic-IR							
TH indices categorized	Q1	Ref	–	Ref	–	Ref	–
	Q2	0.05(0.10)	0.61	0.04(0.11)	0.71	-0.01(0.11)	0.91
	Q3	0.06(0.11)	0.58	0.03(0.11)	0.78	0.12(0.11)	0.28
TH indices continuous	–	0.08(0.11)	0.46	0.08(0.09)	0.41	0.004(0.003)	0.17
Matsuda index							
TH indices categorized	Q1	Ref	–	Ref	–	Ref	–
	Q2	0.07(0.08)	0.37	0.03(0.08)	0.74	-0.002(0.08)	0.98
	Q3	0.03(0.08)	0.69	0.04(0.08)	0.62	-0.07(0.08)	0.34
TH indices continuous	–	0.05(0.07)	0.53	0.03(0.06)	0.69	-0.0005(0.002)	0.80
Adipo-IR							
TH indices categorized	Q1	Ref	–	Ref	–	Ref	–
	Q2	0.15(0.09)	0.09	0.18(0.09)	0.04*	0.10(0.09)	0.26
	Q3	0.20(0.09)	0.03*	0.18(0.09)	0.04*	0.22(0.09)	0.02*
TH indices continuous	–	0.17(0.09)	0.053	0.15(0.08)	0.04*	0.005(0.002)	0.03*

Adjustment for age, sex, body mass index, hypertension, hyperlipidemia, and diabetes.

TH, thyroid hormone; TFQI, Thyroid Feedback Quantile-based Index; TSHI, thyroid stimulating hormone index; TT4RI, thyrotrophic T4 resistance index; HOMA-IR, homeostasis model assessment of insulin resistance; Hepatic-IR, hepatic insulin resistance index; Adipo-IR, adipose tissue insulin resistance index; SE, standard error; Ref, reference.

*P<0.05.

-, Not applicable.

## 4 Discussion

In this study of euthyroid individuals with obesity, we observed a positive but weak correlation between TH sensitivity indices and IR calculated by HOMA-IR, which lost its significance after adjustment for age, sex, BMI, hypertension, hyperlipidemia, and diabetes. We also first found that reduced central TH sensitivity was significantly associated with adipose IR after adjustment for other potential confounders. Our study further confirmed the association between decreased TH sensitivity and metabolic disorders in people with obesity.

TH could regulate some genes involved in gluconeogenesis, glycogen metabolism, and insulin signaling (1). In animal studies, it was observed that the induction of hyperthyroidism or hypothyroidism in mice resulted in corresponding increases or decreases in the hepatocyte membrane glucose transporter (GLUT2) content, highlighting the regulatory role of TH on GLUT2 expression on hepatocyte membranes. TH up-regulates hepatic GLUT2 mRNA and protein expression, increasing hepatic glucose output (27). TH could also modulate hepatic glucose metabolism by a central pathway. Stimulation of TH-sensitive neurons in the paraventricular nucleus in the euthyroid condition increases endogenous glucose production via sympathetic projections to the liver, independently of circulating glucoregulatory hormone concentrations (28). Similarly, thyroid functions also affect GLUT2 expression in pancreatic islets β cells (29). However, although we observed a positive but weak correlation between TH sensitivity indices and IR calculated by HOMA-IR and hepatic-IR (**Figure 1**), the associations lost their significance after adjustment for age, sex, BMI, hypertension, hyperlipidemia, and diabetes, and we didn’t find a significant association between TH sensitivity indices and the Matsuda index. Conversely, one previous study of 80 prepubertal euthyroid children with obesity revealed that TFQI was negatively associated with the Matsuda-index, demonstrating an association between decreased central sensitivity to THs and decreased whole-body insulin sensitivity (19). The populations in that study were Caucasian and it excluded children with impaired fasting blood glucose or impaired glucose tolerance; Our study enrolled Asian adults instead of Caucasian children and was adjusted for different confounding factors, including age, sex, BMI, hypertension, hyperlipidemia, and diabetes. We further conducted stratified analyses and found that sex, hypertension, hyperlipidemia, and diabetes were not significant modifiers in the associations between TH sensitivity and IR. The associations between central TH sensitivity and hepatic and whole-body insulin sensitivity need to be explored in future studies with larger samples and more general populations than just people with obesity.

Our study demonstrated a negative association between central TH sensitivity and adipose IR in people with obesity. TH regulates lipogenesis and lipolysis primarily by modulating adrenergic activity. Reduced sensitivity of TH may lead to a marked reduction in catecholamine-stimulated lipolysis, increased visceral adiposity, and decreased insulin sensitivity (30). The intricate relationship between TH and adipose tissue has always been a hot topic for researchers. T3 induction of lipogenic enzymes which are involved in fatty acid synthesis and lipogenesis could aggravate the dysregulation of liver glucose and lipid metabolism, a characteristic of IR (1). An inactivating mutation in the human type 2 deiodinase(D2) gene could lead to decreased intracellular availability of active thyroid hormone, which in turn, would decrease the transcription of GLUT4 in insulin-sensitive tissues, such as skeletal muscle and adipose tissue, contributing to IR (31). The negative association between central TH sensitivity and adipose IR might also be due to some cytokines. In hyperthyroidism, subcutaneous adipose tissue releases interleukin 6 (IL-6) and tumor necrosis factor-alpha (TNF-α), which could then act as an endocrine mediator of IR in lipolysis (32). Research has shown that TNF-α and IL, specifically IL-1 and IL-6, can suppress the mRNA expression of the sodium/iodide symporter, which is a critical component in the synthesis of THs. In addition to this, proinflammatory cytokines have been linked to the inhibition of type 1 deiodinase (D1) and the stimulation of type 3 deiodinase (D3) in the human hepatocarcinoma cell line (33–35). Another potential mechanism is leptin. Mild hypothyroidism is also characterized by decreased insulin responsiveness in skeletal muscle and adipose tissue, which is in part due to lowered plasma leptin levels and the overexpression of resistin in adipose tissue, and this insulin resistance is partly alleviated by intracerebroventricular leptin administration (36). According to the authors of this study, the dysregulation of leptin action at the hypothalamus acts partly roles in the development of IR. Leptin receptors have been found in the anterior pituitary and thyroid gland. Studies suggest that in the rat pituitary, leptin may act as an autocrine/paracrine inhibitor of TSH release, and also suppresses TSH-induced thyroid function (37, 38). Furthermore, the metabolism of THs can be influenced by leptin. The administration of exogenous leptin has been shown to increase the activity of D1 in the liver and pituitary gland, while concurrently leading to a decrease in the activity of D2 in the hypothalamus and brown adipose tissue (33).

Interestingly, we further did mediation analysis and found that the blood lipids, especially TG levels, mediated the correlation between TH sensitivity indices and adipose IR. The effects of TH sensitivity indices on Adipo-IR were alleviated when conditioning on TG levels. Results of the mediation analysis showed that 6.1% (*P*=0.055) of the effect of TFQI, 6.2% (*P*=0.025) of the effect of TSHI, and 2% (*P*=0.001) of the effect of TT4RI on Adipo-IR could be explained by TG levels. Previous study indicated that plasma TH concentration is associated with hepatic TG content (39). T3 is also capable of modulating the expression of lipogenic enzyme and augments the accumulation of TG in adipocytes (40). The interaction between TH and adiposity is reciprocal. TH exerts significant regulatory effects on the central nervous system. Administering T3 centrally leads to an increase in body temperature, a decrease in hypothalamic AMP-activated protein kinase (AMPK) levels and an enhanced activity in the sympathetic nerves, and upregulates thermogenic markers in brown adipose tissue. The hypothalamic AMPK and fatty-acid metabolism are integral to TH’s regulation of energy balance (41). In primary human differentiated adipocytes, TSH triggers lipolysis and impedes insulin signaling via the inhibition of phosphorylation on protein kinase B (Akt) (42). This mechanism could potentially contribute to the development of IR. However, another study in differentiated adipocytes showed that TSH could directly induce the activity of glycerol-3-phosphate-acyltransferase 3, the rate-limiting enzyme in TG synthesis, leading to an increase in TG synthesis. THs in the liver promote lipogenesis from glucose metabolism and the re-esterification of FFAs into TG by upregulating lipogenic gene transcription. THs also concurrently increase hepatic lipase activity, lipophagy, and mitochondrial fatty acid oxidation—the main mechanisms the liver employs to mitigate steatosis (43). THs increase lipoprotein lipase activity, affecting very low-density lipoprotein (VLDL) levels in the liver and serum, potentially leading to increased serum TG, as the primary lipid component of VLDL is TG (33). In addition, Adipo-IR correlates with TG levels. Adipose tissue dysfunction and insulin resistance escalate lipolysis and FFA release, leading to decreased lipoprotein lipase activity and increased cholesteryl ester transfer protein expression. This dysregulation enhances hepatic TG-rich VLDL production and reduces TG hydrolysis, resulting in hypertriglyceridemia and lipid metabolic disorders (44).

THs display a dual role of both imitating and countering insulin’s actions across varying organs, such as insulin agonistic in muscle or antagonistic in the liver (1). Nevertheless, this interaction maintains a delicate equilibrium, crucial for normal glucose metabolism. An imbalance, either deficiency or surplus of THs, can disrupt this equilibrium, resulting in changes in carbohydrate metabolism. Furthermore, impaired sensitivity to TH was also associated with metabolic disorders, even in the euthyroid population (12, 14–17). In conclusion, our study indicated that impaired central sensitivity to TH was associated with adipose IR in euthyroid people with obesity, and further confirmed the importance of decreased TH sensitivity in metabolic diseases. Our study provides new evidence for the role of reduced central TH sensitivity on glucose and lipid metabolism, laying a foundation for future research on the relationship between THs and consequent cardiovascular metabolic risks.

The present study has some limitations. First, its cross-sectional design does not provide a causal explanation of the relationships. Second, this is a single-center study of subjects with obesity, limiting the generalizability to people with normal weight and people from other countries. Thirdly, the participants in our study were outpatients with obesity who counseled weight loss in the hospital instead of ordinary persons with obesity, which might cause a selection bias. Finally, we didn’t collect data on some potential confounding factors, such as diet, physical activity, and other hormonal influences, which might leave some residual confounding. Future research taking these factors into account and with larger samples and more general populations is needed.

## Data availability statement

The raw data supporting the conclusions of this article will not be made publicly available because the ethical approval obtained for this study prevents the human data being shared publicly to protect patients’ privacy. Requests to access the datasets should be directed to GW, wangguang@bjcyh.com.

## Ethics statement

The studies involving humans were approved by Ethical Review Board of Beijing Chao-Yang Hospital, Capital Medical University. The studies were conducted in accordance with the local legislation and institutional requirements. The participants provided their written informed consent to participate in this study.

## Author contributions

YW: Conceptualization, Data curation, Formal Analysis, Funding acquisition, Investigation, Methodology, Software, Validation, Visualization, Writing – original draft. XL: Conceptualization, Methodology, Writing – original draft. RC: Writing – original draft. JL: Conceptualization, Methodology, Project administration, Supervision, Writing – review & editing. GW: Conceptualization, Funding acquisition, Methodology, Project administration, Resources, Supervision, Writing – review & editing.

## References

[1] Brenta G. Why can insulin resistance be a natural consequence of thyroid dysfunction? J Thyroid Res. (2011) 2011:152850. doi: 10.4061/2011/152850 21941681 PMC3175696

[2] Le TN Celi FS Wickham EP 3rd. Thyrotropin levels are associated with cardiometabolic risk factors in euthyroid adolescents. Thyroid. (2016) 26:1441–9. doi: 10.1089/thy.2016.0055 PMC5067795 27599541

[3] Martinez B Ortiz RM. Thyroid hormone regulation and insulin resistance: insights from animals naturally adapted to fasting. Physiol (Bethesda). (2017) 32:141–51. doi: 10.1152/physiol.00018.2016 28202624

[4] Fernández-Real JM López-Bermejo A Castro A Casamitjana R Ricart W. Thyroid function is intrinsically linked to insulin sensitivity and endothelium-dependent vasodilation in healthy euthyroid subjects. J Clin Endocrinol Metab. (2006) 91:3337–43. doi: 10.1210/jc.2006-0841 16804039

[5] Ferrannini E Iervasi G Cobb J Ndreu R Nannipieri M. Insulin resistance and normal thyroid hormone levels: prospective study and metabolomic analysis. Am J Physiol Endocrinol Metab. (2017) 312:E429–e36. doi: 10.1152/ajpendo.00464.2016 28246105

[6] Spira D Buchmann N Dörr M Markus MRP Nauck M Schipf S. Association of thyroid function with insulin resistance: data from two population-based studies. . Eur Thyroid J. (2022) 11(2):e210063. doi: 10.1530/ETJ-21-0063 35085102 PMC8963174

[7] Ambrosi B Masserini B Iorio L Delnevo A Malavazos AE Morricone L. Relationship of thyroid function with body mass index and insulin-resistance in euthyroid obese subjects. J Endocrinol Invest. (2010) 33:640–3. doi: 10.1007/BF03346663 20339314

[8] Bos MM Smit RAJ Trompet S van Heemst D Noordam R. Thyroid signaling, insulin resistance, and 2 diabetes mellitus: A mendelian randomization study. J Clin Endocrinol Metab. (2017) 102:1960–70. doi: 10.1210/jc.2016-2816 28323940

[9] Choi YM Kim MK Kwak MK Kim D Hong EG. Association between thyroid hormones and insulin resistance indices based on the Korean National Health and Nutrition Examination Survey. Sci Rep. (2021) 11:21738. doi: 10.1038/s41598-021-01101-z 34741077 PMC8571382

[10] Temizkan S Balaforlou B Ozderya A Avci M Aydin K Karaman S. Effects of thyrotrophin, thyroid hormones and thyroid antibodies on metabolic parameters in a euthyroid population with obesity. Clin Endocrinol (Oxf). (2016) 85:616–23. doi: 10.1111/cen.13095 27150556

[11] Amouzegar A Kazemian E Gharibzadeh S Mehran L Tohidi M Azizi F. Association between thyroid hormones, thyroid antibodies and insulin resistance in euthyroid individuals: A population-based cohort. Diabetes Metab. (2015) 41:480–8. doi: 10.1016/j.diabet.2015.04.004 26049821

[12] Laclaustra M Moreno-Franco B Lou-Bonafonte JM Mateo-Gallego R Casasnovas JA Guallar-Castillon P. Impaired sensitivity to thyroid hormones is associated with diabetes and metabolic syndrome. Diabetes Care. (2019) 42:303–10. doi: 10.2337/dc18-1410 30552134

[13] Jostel A Ryder WD Shalet SM. The use of thyroid function tests in the diagnosis of hypopituitarism: definition and evaluation of the TSH Index. Clin Endocrinol (Oxf). (2009) 71:529–34. doi: 10.1111/j.1365-2265.2009.03534.x 19226261

[14] Sun Y Teng D Zhao L Shi X Li Y Shan Z. Impaired sensitivity to thyroid hormones is associated with hyperuricemia, obesity, and cardiovascular disease risk in subjects with subclinical hypothyroidism. Thyroid. (2022) 32:376–84. doi: 10.1089/thy.2021.0500 35078326

[15] Ding X Wang Y Liu J Wang G. Impaired sensitivity to thyroid hormones is associated with elevated homocysteine levels in the euthyroid population. J Clin Endocrinol Metab. (2022) 107:e3731–e7. doi: 10.1210/clinem/dgac371 35708733

[16] Sun H Zhu W Liu J An Y Wang Y Wang G. Reduced sensitivity to thyroid hormones is associated with high remnant cholesterol levels in chinese euthyroid adults. J Clin Endocrinol Metab. (2022) 108:166–74. doi: 10.1210/clinem/dgac523 36071542

[17] Mehran L Delbari N Amouzegar A Hasheminia M Tohidi M Azizi F. Reduced sensitivity to thyroid hormone is associated with diabetes and hypertension. J Clin Endocrinol Metab. (2022) 107:167–76. doi: 10.1210/clinem/dgab646 34480566

[18] Ruderman NB Carling D Prentki M Cacicedo JM. AMPK. insulin resistance, and the metabolic syndrome. J Clin Invest. (2013) 123:2764–72. doi: 10.1172/JCI67227 PMC3696539 23863634

[19] Corica D Licenziati MR Calcaterra V Currò M Di Mento C Curatola S. Central and peripheral sensitivity to thyroid hormones and glucose metabolism in prepubertal children with obesity: pilot multicenter evaluation. Endocrine. (2023) 80:308–11. doi: 10.1007/s12020-022-03276-5 36484935

[20] Matthews DR Hosker JP Rudenski AS Naylor BA Treacher DF Turner RC. Homeostasis model assessment: insulin resistance and beta-cell function from fasting plasma glucose and insulin concentrations in man. Diabetologia. (1985) 28:412–9. doi: 10.1007/BF00280883 3899825

[21] Abdul-Ghani MA Matsuda M Balas B DeFronzo RA. Muscle and liver insulin resistance indexes derived from the oral glucose tolerance test. Diabetes Care. (2007) 30:89–94. doi: 10.2337/dc06-1519 17192339

[22] Matsuda M DeFronzo RA. Insulin sensitivity indices obtained from oral glucose tolerance testing: comparison with the euglycemic insulin clamp. Diabetes Care. (1999) 22:1462–70. doi: 10.2337/diacare.22.9.1462 10480510

[23] Gastaldelli A Harrison SA Belfort-Aguilar R Hardies LJ Balas B Schenker S. Importance of changes in adipose tissue insulin resistance to histological response during thiazolidinedione treatment of patients with nonalcoholic steatohepatitis. Hepatology. (2009) 50:1087–93. doi: 10.1002/hep.23116 19670459

[24] Zhou B. Predictive values of body mass index and waist circumference to risk factors of related diseases in Chinese adult population. Zhonghua Liu Xing Bing Xue Za Zhi. (2002) 23:5–10. doi: 10.3760/cma.j.issn.0254-6450.2002.01.103 12015100

[25] Zhu J Gao R Zhao S Lu G Zhao D Li J. 2016 Chinese guidelines for the management of dyslipidemia in adults. J Geriatr Cardiol. (2018) 15:1–29. doi: 10.11909/j.issn.1671-5411.2018.01.011 29434622 PMC5803534

[26] American Diabetes Association Professional Practice Committee. 2. Classification and diagnosis of diabetes: standards of medical care in diabetes-2022. Diabetes Care. (2022) 45:S17–s38. doi: 10.2337/dc22-S002 34964875

[27] Sun B Chen H Xue J Li P Fu X. The role of GLUT2 in glucose metabolism in multiple organs and tissues. Mol Biol Rep. (2023) 50:6963–74. doi: 10.1007/s11033-023-08535-w PMC10374759 37358764

[28] Klieverik LP Janssen SF van Riel A Foppen E Bisschop PH Serlie MJ. Thyroid hormone modulates glucose production via a sympathetic pathway from the hypothalamic paraventricular nucleus to the liver. Proc Natl Acad Sci United States America. (2009) 106:5966–71. doi: 10.1073/pnas.0805355106 PMC2660059 19321430

[29] Gholami H Jeddi S Zadeh-Vakili A Farrokhfall K Rouhollah F Zarkesh M. Transient congenital hypothyroidism alters gene expression of glucose transporters and impairs glucose sensing apparatus in young and aged offspring rats. Cell Physiol Biochem. (2017) 43:2338–52. doi: 10.1159/000484386 29073628

[30] Liu YY Schultz JJ Brent GA. A thyroid hormone receptor alpha gene mutation (P398H) is associated with visceral adiposity and impaired catecholamine-stimulated lipolysis in mice. J Biol Chem. (2003) 278:38913–20. doi: 10.1074/jbc.M306120200 12869545

[31] Mentuccia D Proietti-Pannunzi L Tanner K Bacci V Pollin TI Poehlman ET. Association between a novel variant of the human type 2 deiodinase gene Thr92Ala and insulin resistance: evidence of interaction with the Trp64Arg variant of the beta-3-adrenergic receptor. Diabetes. (2002) 51:880–3. doi: 10.2337/diabetes.51.3.880 11872697

[32] Mitrou P Boutati E Lambadiari V Tsegka A Raptis AE Tountas N. Insulin resistance in hyperthyroidism: the role of IL6 and TNF alpha. Eur J endocrinology. (2010) 162:121–6. doi: 10.1530/EJE-09-0622 19837795

[33] Teixeira P Dos Santos PB Pazos-Moura CC. The role of thyroid hormone in metabolism and metabolic syndrome. Ther Adv Endocrinol Metab. (2020) 11:2042018820917869. doi: 10.1177/2042018820917869 32489580 PMC7238803

[34] Jakobs TC Mentrup B Schmutzler C Dreher I Köhrle J. Proinflammatory cytokines inhibit the expression and function of human type I 5'-deiodinase in HepG2 hepatocarcinoma cells. Eur J endocrinology. (2002) 146:559–66. doi: 10.1530/eje.0.1460559 11916626

[35] Boelen A Kwakkel J Alkemade A Renckens R Kaptein E Kuiper G. Induction of type 3 deiodinase activity in inflammatory cells of mice with chronic local inflammation. Endocrinology. (2005) 146:5128–34. doi: 10.1210/en.2005-0608 16150911

[36] Cettour-Rose P Theander-Carrillo C Asensio C Klein M Visser TJ Burger AG. Hypothyroidism in rats decreases peripheral glucose utilisation, a defect partially corrected by central leptin infusion. Diabetologia. (2005) 48:624–33. doi: 10.1007/s00125-005-1696-4 15756538

[37] Ortiga-Carvalho TM Oliveira KJ Soares BA Pazos-Moura CC. The role of leptin in the regulation of TSH secretion in the fed state: *in vivo* and *in vitro* studies. J Endocrinol. (2002) 174:121–5. doi: 10.1677/joe.0.1740121 12098670

[38] Isozaki O Tsushima T Nozoe Y Miyakawa M Takano K. Leptin regulation of the thyroids: negative regulation on thyroid hormone levels in euthyroid subjects and inhibitory effects on iodide uptake and Na+/I- symporter mRNA expression in rat FRTL-5 cells. Endocr J. (2004) 51:415–23. doi: 10.1507/endocrj.51.415 15351798

[39] Bril F Kadiyala S Portillo Sanchez P Sunny NE Biernacki D Maximos M. Plasma thyroid hormone concentration is associated with hepatic triglyceride content in patients with type 2 diabetes. J Investig Med. (2016) 64:63–8. doi: 10.1136/jim-2015-000019 26755815

[40] Jiang W Miyamoto T Kakizawa T Sakuma T Nishio S Takeda T. Expression of thyroid hormone receptor alpha in 3T3-L1 adipocytes; triiodothyronine increases the expression of lipogenic enzyme and triglyceride accumulation. J Endocrinol. (2004) 182:295–302. doi: 10.1677/joe.0.1820295 15283690

[41] López M Varela L Vázquez MJ Rodríguez-Cuenca S González CR Velagapudi VR. Hypothalamic AMPK and fatty acid metabolism mediate thyroid regulation of energy balance. Nat Med. (2010) 16:1001–8. doi: 10.1038/nm.2207 PMC2935934 20802499

[42] Felske D Gagnon A Sorisky A. Interacting effects of TSH and insulin on human differentiated adipocytes. Horm Metab Res. (2015) 47:681–5. doi: 10.1055/s-0034-1395673 25502943

[43] Sinha RA Singh BK Yen PM. Direct effects of thyroid hormones on hepatic lipid metabolism. Nat Rev Endocrinol. (2018) 14:259–69. doi: 10.1038/nrendo.2018.10 PMC6013028 29472712

[44] van de Woestijne AP Monajemi H Kalkhoven E Visseren FL. Adipose tissue dysfunction and hypertriglyceridemia: mechanisms and management. Obes Rev. (2011) 12:829–40. doi: 10.1111/j.1467-789X.2011.00900.x 21749607

