# Supplementary material for: Associations between sensitivity to thyroid hormones and insulin resistance in euthyroid adults with obesity
Source: Front Endocrinol (Lausanne). 2024 Aug 8;15:1366830. doi: 10.3389/fendo.2024.1366830 (PMC11338882; doi:10.3389/fendo.2024.1366830)
Supplement: Supplementary file 1 [file Table_1.docx]

Supplementary Material

**
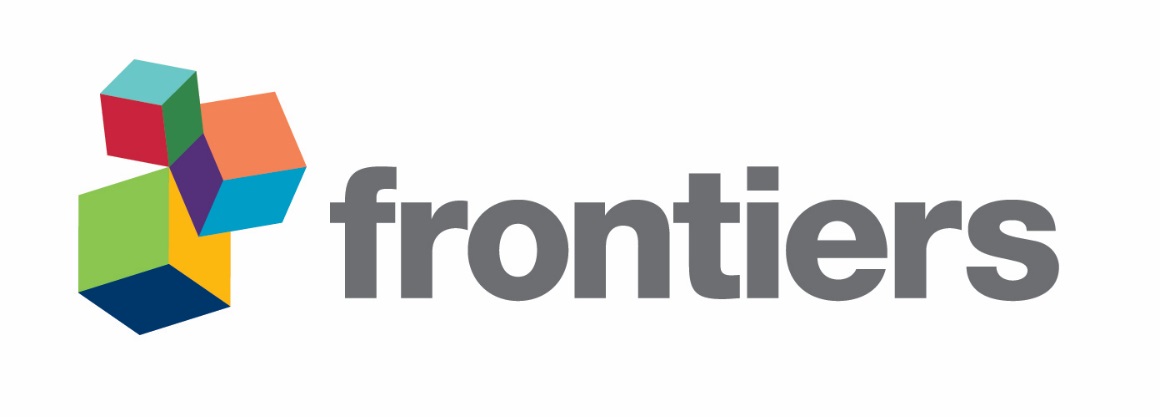
**

**Supplementary Table 1. Tertiles of thyroid hormone sensitivity indices**

|  | Q1 | Q2 | Q3 |
| --- | --- | --- | --- |
| TFQI | [-0.925, -0.204]  n=98 | (-0.204, 0.237]  n=97 | (0.237, 0.915]  n=98 |
| TSHI | [1.37, 2.77]  n=98 | (2.77, 3.21]  n=97 | (3.21, 4.23]  n=98 |
| TT4RI | [8.37, 32.7]  n=98 | (32.7, 45.8]  n=97 | (45.8, 82.9]  n=98 |

Abbreviations: TFQI: Thyroid Feedback Quantile-based Index, TSHI: thyroid stimulating hormone index, TT4RI: thyrotrophic T4 resistance index.

**Supplementary Table 2. Insulin resistance indices across tertiles of thyroid hormone sensitivity indices**

|  | TFQI | | |  | TSHI | | |  | TT4RI | | |
| --- | --- | --- | --- | --- | --- | --- | --- | --- | --- | --- | --- |
|  | Q1 | Q2 | Q3 |  | Q1 | Q2 | Q3 |  | Q1 | Q2 | Q3 |
| HOMA-IR | 7.66 (4.76) | 8.73 (6.10) | 10.4 (8.33) |  | 7.29 (4.16) | 9.44 (6.79) | 10.0 (8.09) |  | 7.67 (5.19) | 8.96 (6.39) | 10.1 (7.88) |
| Hepatic-IR | 155 (79.2) | 174 (100) | 181 (92.4) |  | 152 (71.0) | 180 (107) | 177 (92.0) |  | 148 (74.4) | 172 (86.7) | 192 (108) |
| Matsuda index | 34.8 (27.2) | 37.8 (30.2) | 33.0 (23.6) |  | 34.2 (23.9) | 39.0 (36.0) | 32.4 (18.4) |  | 36.9 (28.1) | 37.6 (33.0) | 31.1 (17.9) |
| Adipo-IR | 16.9 (10.3) | 21.1 (15.4) | 24.7 (18.0) |  | 16.7 (10.4) | 22.1 (15.7) | 23.9 (17.8) |  | 17.0 (11.4) | 21.1 (15.2) | 24.6 (17.6) |

Abbreviations: TFQI: Thyroid Feedback Quantile-based Index, TSHI: thyroid stimulating hormone index, TT4RI: thyrotrophic T4 resistance index, HOMA-IR: homeostasis model assessment of insulin resistance, Hepatic-IR: hepatic insulin resistance index, Adipo-IR: adipose tissue insulin resistance index. Data are shown as mean (standard deviation).
